# RETRACTION: Influence of Environmental Risk Factors on the Development of Wounds Associated with Squamous Cell Carcinoma

**DOI:** 10.1111/iwj.70475

**Published:** 2025-04-02

## Abstract

**RETRACTION:** JiaY. , ChenF. , YanT. , ZhangS. , SalemM. M. , SinghS. , Salem‐BekhitM. M. , KumarS. K. , and AliM. M. , “Influence of Environmental Risk Factors on the Development of Wounds Associated with Squamous Cell Carcinoma,” International Wound Journal 21, no. 3 (2024): e14506, 10.1111/iwj.14506.38010070 PMC10898377

The above article, published online on 27 November 2023, in Wiley Online Library (http://onlinelibrary.wiley.com/), has been retracted by agreement between the journal Editor in Chief, Professor Keith Harding; and John Wiley & Sons Ltd. Following an investigation by the publisher, all parties have concluded that this article was accepted solely on the basis of a compromised peer review process. The editors have therefore decided to retract the article. The authors did not indicate their agreement with the retraction.

**RETRACTION:** Y. Jia , F. Chen , T. Yan , S. Zhang , M. M. Salem , S. Singh , M. M. Salem‐Bekhit , S. K. Kumar , and M. M. Ali , “Influence of Environmental Risk Factors on the Development of Wounds Associated with Squamous Cell Carcinoma,” International Wound Journal 21, no. 3 (2024): e14506, 10.1111/iwj.14506.38010070 PMC10898377

The above article, published online on 27 November 2023, in Wiley Online Library (http://onlinelibrary.wiley.com/), has been retracted by agreement between the journal Editor in Chief, Professor Keith Harding; and John Wiley & Sons Ltd. Following an investigation by the publisher, all parties have concluded that this article was accepted solely on the basis of a compromised peer review process. The editors have therefore decided to retract the article. The authors did not indicate their agreement with the retraction.

